# Attitudes and Beliefs around the Value of Vaccination in the United States

**DOI:** 10.3390/vaccines10091470

**Published:** 2022-09-05

**Authors:** Sarah Childers-Strawbridge, Amanda L. Eiden, Mawuli K. Nyaku, Alexandra A. Bhatti

**Affiliations:** Merck & Co., Inc., Rahway, NJ 07065, USA

**Keywords:** accessibility, attitudes, beliefs, health policy, health records, immunization program, immunization records, life course vaccination, perceptions, policy, vaccines, vaccination, vaccinator

## Abstract

Despite the benefits of immunization, differences in attitudes persist toward vaccines. We captured individuals’ perceptions of vaccines and vaccination across the United States (US) to inform vaccine-related policy development. A survey was completed by 5000 respondents from 10 states. Respondents were screened for inclusion, which included individuals ≥ 18 years of age that had received a vaccine or were unvaccinated but indicated a favorable or neutral attitude towards vaccinations. Participants were excluded if they indicated they did not support the idea of vaccinations. Questions explored perceptions of vaccines for all age groups. Among unvaccinated individuals, the most common concerns were about safety (38%). Most respondents (95%) highlighted the importance of state immunization programs for disease prevention. Access to health and immunization records and immunization information systems were important to 96% and 88% of respondents, respectively, for future health planning. Doctors and healthcare professionals (HCPs) were considered trusted sources for vaccine information (95%). Overall, respondents recognized the importance of vaccination, but documented concerns among the unvaccinated indicated a need for greater promotion regarding vaccine safety. Doctors and HCPs, as trusted information sources, should continue to and increasingly advocate for the importance of immunization to increase vaccine uptake.

## 1. Introduction

Worldwide immunization has made a critical contribution to the reduction of infectious diseases and their associated burden on healthcare systems and national economies throughout a population’s lifetime [1,2]. The United States (US) Centers for Disease Control and Prevention (CDC) estimates that routine vaccination of children born between 1994 and 2018 will prevent 26.8 million hospitalizations, prevent 936,000 deaths, and save almost $1.9 trillion US dollars (USD) in total societal costs (both direct costs to the healthcare system and indirect costs such as loss of productivity) [1,3]. Routine vaccination has improved quality of life and increased both education and labor force participation, which has translated into increased productivity gains across age groups [4,5,6,7]. Adults in developed countries aged ≥65 years accounted for 90% of pneumonia- and influenza-related deaths, indicating the need for continued uptake of vaccines throughout all stages of life [7,8,9]. Additionally, the influenza vaccine has been linked to a reduced risk of acute stroke in a recent large case-control study, suggesting that immunization may confer indirect benefits in the reduction of other diseases [10]. Furthermore, COVID-19 vaccines have played a central role in controlling the COVID-19 pandemic, underscoring the value of vaccines and vaccination [11].

However, despite the benefits of immunization, disparities persist in both the accessibility and provision of vaccines [12]. Understanding perceptions of vaccines and vaccination within target populations is paramount to addressing these disparities, particularly with the recent rise of vaccine hesitancy in the US associated with growing dis- and misinformation through social media and the distrust of governments [13,14,15]. In the US, in 2016, vaccination was considered to be important and beneficial for health by 72% of individuals based on a cross-sectional study [16]. However, approximately 10% of individuals indicated concerns about the risks of vaccination and would not support routine childhood vaccination mandates for school entry [16]. Similarly, a global systematic review including 38 studies reported that 75% of individuals would have their children receive all childhood vaccines [17]. For adults, vaccination coverage rates for most routinely recommended vaccines remains below the Healthy People 2020 objectives for the US; for example, the goal for the influenza vaccine was 80% for adults aged 18–64 years and 90% for adults over 65 years, however, vaccination coverage among adults aged 50–64 years and those 65 years and older was 48% and 72%, respectively; for the pneumococcal vaccine the target was 60% for ages 18–64 at increased risk and 90% in adults over 65 years but coverage rates only reached an estimated 23% of adults aged 19–64 years at increased risk and 69% of adults aged 65 years and over [18,19]. Therefore, while the vaccine uptake for pediatric populations is high, this has not translated to adult populations where the vaccine uptake remains low.

It is important to understand the perceptions, values, and beliefs that drive vaccination choices to inform vaccine policy and programmatic practices to achieve and sustain high vaccination coverage rates for vaccine-preventable diseases [20]. Therefore, the aim of this study was to understand how the US adult population accesses vaccines, the value they place on vaccines and vaccination across their life-course, and the perceived importance of having access to health and immunization records. The findings from this study may be used to support evidence-based vaccine policy and programmatic practices.

## 2. Materials and Methods

We report on an online survey administered in August 2021 to assess the perceived value of vaccines and vaccination among the US adult population. Respondents were ≥18 years of age and must either have received a vaccine for a vaccine-preventable disease or were unvaccinated but were not fully vaccine-hesitant or had an unfavorable attitude towards vaccines. Survey respondents were not eligible for inclusion if they had never received a vaccine and were also opposed to vaccines. The full survey screener can be found in the supplementary materials. The survey took respondents approximately 20 min to complete and was mobile-friendly, enabling respondents to participate in the study using a range of devices including desktop computers, notebooks, laptops, tablets, or smartphones.

Respondents were recruited through market research panels, with a target of 5000 total respondents (Figure S1). States were grouped into five regions for analysis: Northeast (New Jersey and Pennsylvania), South (Tennessee and North Carolina), South-Central (Louisiana and Oklahoma), Midwest (Ohio and Illinois), and West (Nevada and Idaho). States were selected based on the presence of existing or forthcoming legislative threats to vaccination as assessed by the researchers. Legislative threats were defined as any policy that would hinder or block access to vaccination, threaten the systems that support vaccination including delivery of vaccines, or create doubt or question the safety and efficacy of vaccines.

Respondents were presented with a series of closed-ended questions for which they selected an answer from a list of prespecified responses. Most responses to questions took the form of five-point Likert scales, where participants were asked to select their responses on a scale. Most questions without a Likert scale were multiple choice. Two questions allowed respondents to type in free text, however, these questions explored the impact of COVID-19 on perceptions of vaccination. The results from all COVID-19-related questions can be found in the supplementary files. Data collected included: (1) perceptions of vaccines and immunization programs in general, (2) perceptions of vaccines and immunization programs for specific age groups, (3) factors influencing vaccine choices, (4) levels of confidence in the science behind vaccines, (5) perceived importance of access to health and immunization records and state immunization information systems (IIS), (6) sources most trusted for vaccine information, and (7) preferences regarding the location of vaccine administration. Most survey questions considered vaccines in general for all populations, with some specifying a particular age group. The full survey can be viewed in the supplementary materials. No statistical analyses were conducted.

## 3. Results

### 3.1. Respondent Characteristics

A total of 5000 respondents completed the survey, comprised of 500 from each of the 10 states (New Jersey, Pennsylvania, Ohio, Illinois, Tennessee, North Carolina, Louisiana, Oklahoma, Nevada, and Idaho). Overall, 43% of the population were male and 56% were female (1% selected “other” and <1% selected “prefer not to say”) with a mean age of 53 years. There were 1241 (25%) respondents with children living in their household and 3759 (75%) respondents without children in the household. Ninety-five percent had received a vaccination for a vaccine-preventable disease in their lifetime (this included COVID-19 vaccines), and 91% of parents had their children receive a vaccination for a vaccine-preventable disease (Table S1).

### 3.2. Vaccine Perceptions

#### 3.2.1. General Perceptions Regarding Vaccinations and the Value of State Immunization Programs

**Figure 1:**
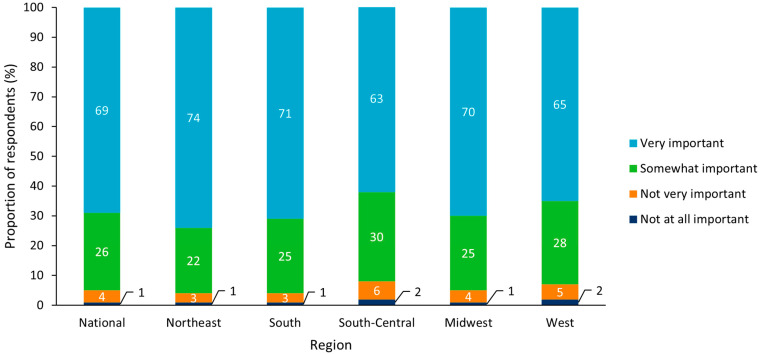
Perceived importance of state vaccination programs for all respondents (n = 5000) Results split by US region.

Across all regions, 95% believed that a state-wide immunization program was “very” or “somewhat” important, with the highest level of agreement in importance in the Northeast (96%) and the lowest in South-Central (93%; Figure 1). At the state level, the highest level of agreement that vaccination programs are important was in New Jersey, Nevada, North Carolina, and Tennessee with 96% from each state and the lowest was in Idaho with 90%. When asked about their agreement with statements regarding vaccines, 95% either “agreed” or “strongly agreed” with the statement “Vaccines help prevent disease for children” from a clinical perspective, and 93% either “agreed” or “strongly agreed” with the statement “It is cheaper to prevent disease than treat it” from an economic perspective. Of the respondents in the Northeast, 97% and 95% either “agreed” or “strongly agreed” with these two statements, respectively, and of the respondents in the West and South-Central regions, 94% and 91% either “agreed” or “strongly agreed” with these two statements, respectively.

**Figure 2:**
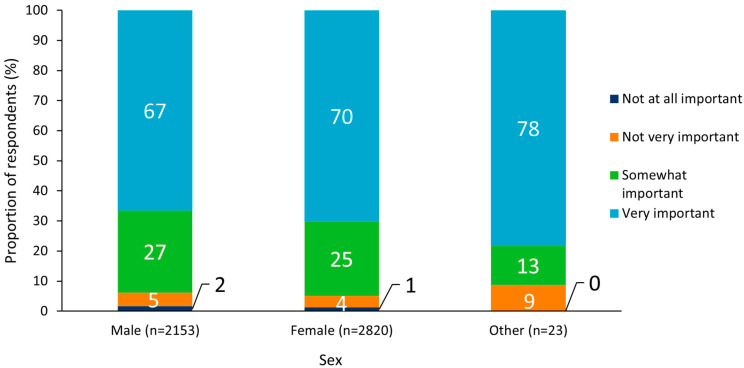
Perceived importance of state vaccination programs split by respondent sex.

**Figure 3:**
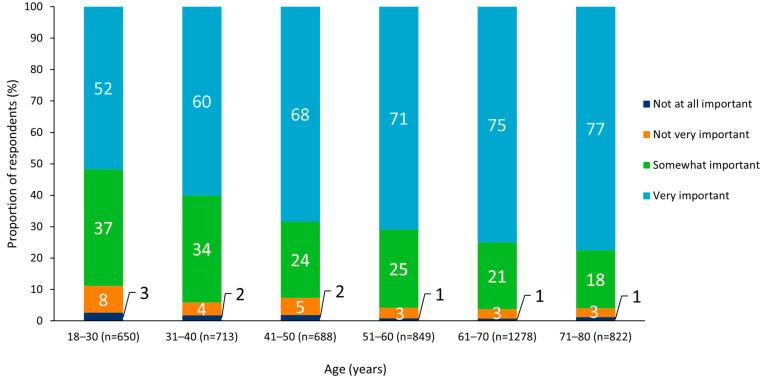
Perceived importance of state vaccination programs split by respondent age group.

**Figure 4:**
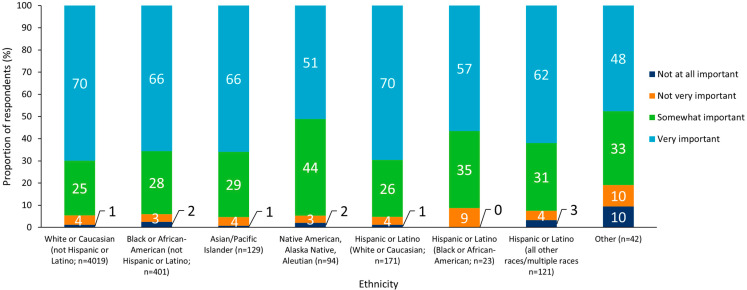
Perceived importance of state vaccination programs split by respondent ethnicity.

An analysis of results by subgroup revealed no notable differences between sexes, but there was a general trend whereby state-wide immunization programs were more often viewed as “very” or “somewhat” important with increasing respondent age (Figure 2 and Figure 3). Respondents who identified as Hispanic or Latino (White or Caucasian) were most likely to report that state-wide immunization programs were “very” or “somewhat” important (96%), and those who reported their ethnicity as “other” were least likely to perceive immunization programs as “very” or “somewhat” important (81%; Figure 4).

Across the 10 states, 88% reported that they were “very” or “somewhat” confident in the science behind vaccines, with the highest proportion who were “very” or “somewhat” confident in the Northeast (92%), and the lowest in the West (84%). On a state level, respondents in New Jersey were most often “very” or “somewhat” confident (93%) and respondents in Idaho least often reported being “very” or “somewhat” confident in the science behind vaccines (82%). 

All respondents who reported that they had not received a vaccine but did not have a completely unfavorable opinion of vaccines (n = 194) were asked to record whether they agreed with possible factors influencing their vaccine choices. The statements which respondents most often agreed with were “I have concerns about vaccine safety” (38%) and “I need more information about vaccines” (22%). The statements least often selected as concerns by respondents were “I do not have easy access to vaccines (due to location or transportation)” and “I don’t know if my insurance covers vaccines”, which were concerns for only 3% and 4% of individuals, respectively.

#### 3.2.2. Perceptions of Vaccines for Specific Age Groups

Respondents reported that state immunization programs were relatively important for all age groups from 0 years to ≥65 years of age (Table 1). Although the State Department of Health manages the programs for all immunizations across age groups, respondents were asked for their perceptions of vaccines to support specific age group populations. Across all 10 states, state immunization programs that support vaccination were considered either “somewhat” or “very” important by 92% of participants, on average, for all age groups, with the highest percentage for children aged 9–12 years at 95%. Notably, immunization programs for older adults aged ≥65 years were overall more often considered “somewhat” or “very” important at 93% compared to immunization programs for babies aged 0–2 years with 89%. Overall, the percentage of respondents reporting “somewhat” or “very” important was consistent within different age groups, across all regions, with no more than ±2% variation.

When parents were asked about immunization programs for their own children, 96% reported that having their children immunized was “somewhat” or “very” important. Respondents in the Northeast most often reported that this was “somewhat” or “very” important at 98% compared to 95% reported by the respondents in the South-Central, Midwest, and West regions. On a state level, the greatest proportion that considered vaccinating their children to be “somewhat” or “very” important was in New Jersey and Tennessee with 98% in both states, and the lowest proportion was in Louisiana with 94%.

There were 77 respondents who were not opposed to vaccines but whose children had not received a vaccine. The most common factors that influenced why respondents’ children were not vaccinated were “I need more information about vaccines” and “I have concerns about vaccine safety”. Across all regions, these statements were selected as contributing factors by 25% and 22%, respectively.

### 3.3. Access to Information and Immunization Records

Across all 10 states, 96% reported that access to health records was “somewhat” or “very” important for future health planning, with the greatest proportion reporting that access was “somewhat” or “very” important in the Northeast (97%) and the lowest proportion in the South-Central region (94%). At the state level, the greatest proportion reporting that access was “somewhat” or “very” important was observed in New Jersey (97%) and the lowest proportion in Louisiana (93%). 

Access to immunization records was also important, with 94% reporting that access was “somewhat” or “very” important for future health planning. Perceptions were similar across regions with 93% in the South-Central and West to 95% in the Northeast and Midwest. By state, the greatest proportion who selected “somewhat” or “very” important was 96% in Illinois and the lowest was 92% in Louisiana.

**Figure 5:**
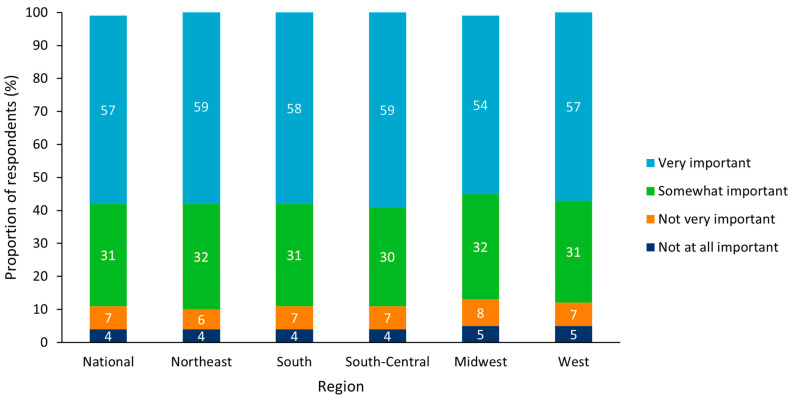
Perceived importance of immunization information systems (IIS) for all respondents without religious objections to vaccination (n = 4917). Results split by US region.

Most respondents reported that state IIS were “somewhat” or “very” important (88%), with the greatest proportion reporting that state IIS were “somewhat” or “very” important in the Northeast (91%) and the lowest in the Midwest (86%) (Figure 5); less than 6% across all regions found IIS to be “not at all important”. On a state level, New Jersey had the highest proportion (94%) that considered IIS “somewhat” or “very” important and Idaho had the lowest proportion (85%). When presented with the statement “Immunization (vaccination) records and immunization information systems (IIS) play an important role in managing and stopping the spread of diseases that can be prevented by getting vaccines”, 88% reported that they “agreed” or “strongly agreed”. There was little variation across regions (ranging from 90% in the Northeast to 87% in the Midwest and West). On a state level, the greatest proportion who “agreed” or “strongly agreed” was in New Jersey (93%) and the lowest proportion was in Idaho (84%).

### 3.4. Trust in Healthcare Providers

**Figure 6:**
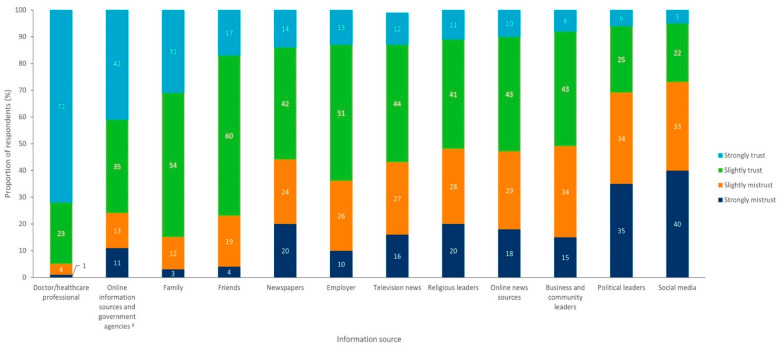
Level of trust in vaccine information sources for all respondents (n = 5000). ^a^ For example, the Centers for Disease Control and Prevention (CDC).

Across the 10 states, 95% of respondents reported that they received and trusted information on vaccines from their doctors or a healthcare professional (Figure 6) and were less likely to receive or trust such information from social media or political leaders (trusted by 27% and 31%, respectively). Across regions and states, there was little variation in the trust respondents placed in their doctor or healthcare professional (94–96% trusted these individuals as sources), but respondents in the South-Central region were more likely to trust social media (32%) than respondents from other regions, which ranged from 22 to 27%.

### 3.5. Access to a Vaccinator

**Figure 7:**
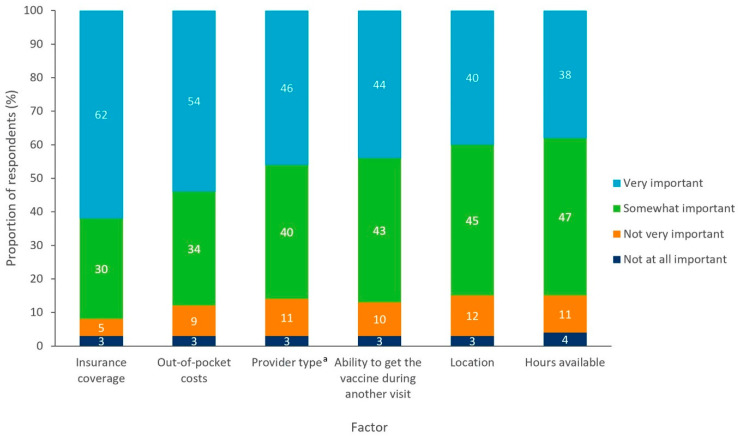
Factors influencing the receipt of a vaccine for all respondents without religious objections (n = 4917). ^a^ Provider type referred to whether vaccines are administered by a primary care doctor, specialist doctor, pharmacist, etc.

Respondents reported that insurance coverage and potential out-of-pocket costs were the most important factors in receiving a vaccine, which was considered either “somewhat” or “very important” by 92% and 88%, respectively (Figure 7). These findings were consistent across regions and states. The time of day that vaccination services were provided was the least important factor for respondents, as it was considered either “somewhat” or “very important” by 85% of respondents.

The most common location for individuals to receive vaccines was at a doctor’s office (64%) and the second most common location was at a local pharmacy (39%). Results were relatively consistent across regions and states, although respondents in the South-Central area were more likely to be vaccinated in a community clinic (17%), school clinic (4%), employer-based clinic (8%), or hospital (15%) than respondents from any other region. However, the doctor’s office and local pharmacy were still the most used locations in this region, with 61% and 34%, respectively. Similar results were observed across regions when respondents were asked specifically about locations for their children to receive vaccines, although a doctor’s office was reported more frequently than a local pharmacy (68% and 22%, respectively). The doctor’s office was used most often across all regions (range, 63% in the South-Central region to 81% in the South) and states (range, 59% in Oklahoma to 88% in North Carolina). 

## 4. Discussion

Research has shown that vaccination rates for CDC-recommended vaccines for all age groups have declined during the COVID-19 pandemic due to range of factors including, fear of infection with SARS-CoV-2, insurance coverage issues due to job loss, re-prioritization of health services, logistical barriers, and adherence to “stay-at-home” measures to prevent the spread of infection [21,22,23,24,25]. This underscores the importance of actionable insights that can help improve vaccine uptake to prevent the resurgence of vaccine-preventable diseases as a result of the COVID-19 pandemic. Our study provides an overview of the perceptions of vaccines and vaccination among the US population, generating insights that can be leveraged to drive vaccine uptake and improve coverage across the life-course.

Overall, most respondents considered state-wide immunization programs for both adults and children to be important, although vaccination for children aged 9–12 years was considered the most important. Further research should be conducted to understand the reason for this perception. Analysis by respondent age group showed that state-wide immunization programs were generally perceived to be more important with increasing age. Previous publications have documented a greater vaccine acceptability among older adults compared to younger adults, likely due to increased susceptibility to severe disease [26,27,28]. These findings suggest that vaccine education programs would be most beneficial when targeted toward younger adults to reduce vaccine hesitancy. Among respondents who had never received a vaccination, concerns about vaccine safety align with previous research and should be addressed with greater availability of information on the monitoring mechanisms for adverse events following vaccination and rigorous safety protocols [29,30]. The development of tailored vaccination education materials to match the needs of individual communities and subpopulations has been an effective approach to allay the growing hesitancy and concerns towards vaccines and vaccination services [31]. Regional variations in the survey highlight areas to target with tailored vaccination education initiatives to increase understanding of vaccine safety. The fact that most respondents viewed state-wide immunization programs as important is a positive finding for future vaccine uptake, as state immunization programs have been proven to help address vaccine uptake disparities and maintain high vaccination coverage rates [32,33,34]. 

Most respondents indicated that having access to health and immunization records was important for future health planning, such as receiving future vaccines. Additionally, almost all respondents agreed that IIS plays an important role in controlling and preventing the spread of vaccine-preventable diseases. This supports previous findings that access to health information empowers patients to make informed vaccination decisions and reduces missed opportunities for vaccination [35,36,37]. Therefore, increased transparency regarding health and immunization records may contribute to increasing the acceptance of vaccines and increasing the levels of timely vaccine uptake. The development of digital tools for patients has been accelerated during the COVID-19 pandemic, which can serve as a foundation to extend such tools for all CDC-recommended vaccinations [38,39].

Our study also found that doctors and other healthcare professionals remained the most trusted sources of vaccine information. These findings are consistent with previous reports that individuals in the US place the greatest trust in doctors as sources of vaccine information [40,41]. This is important given the rise in mis- and disinformation across digital and mainstream media channels, particularly in relation to COVID-19 vaccines during the pandemic as this mis- and disinformation has been linked to vaccine hesitancy [42]. 

As trusted messengers, healthcare professionals are uniquely positioned to play a critical role in building confidence in vaccines, along with the science behind them. This is critical as this study showed that only 45% of respondents were “very” confident in the science behind vaccines, and lack of information and safety concerns were key drivers of the decision to not receive a vaccine. Therefore, although healthcare professionals were the most trusted sources of vaccine information, the ease of access to misinformation in digital and social media may still influence vaccination perceptions and choices despite these channels being reported as less trusted sources of information [43,44,45].

These findings are reflective of published literature, particularly in the COVID-19 era where vaccine-related misinformation is highly prevalent [46]. This has undermined public trust and resulted in a substantial proportions of US residents who are unwilling to receive a COVID-19 vaccine [47]. Concerns about safety are well-documented barriers to the receipt of vaccination, and previous research has suggested that a tailored approach is required to address these specific issues and communicate the value of vaccination [45,46]. Targeted educational strategies would additionally alleviate common concerns about a lack of information on vaccines and may allow vaccine-related mis- and disinformation to be addressed and corrected [48,49].

Amongst access-related factors, having insurance coverage and clarity on potential out-of-pocket costs were the main factors influencing receiving a vaccination. This supports previous findings that cost is a barrier to vaccine access in the US, highlighting the critical need to eliminate any out-of-pocket costs that still exist for some patients [21,50]. These findings are also indicative of the requirement for better education for the public on how vaccines are covered by insurance providers and that vaccines are also often made available to uninsured individuals [34,51]. There are policies currently in place that have greatly reduced barriers to vaccine coverage by reducing out-of-pocket costs and improving access across the US, including the Affordable Care Act (ACA), Vaccines for Children program (VFC), Section 317 of the Public Health Service Act; the latter of which authorizes the federal purchase of vaccines for discreet populations such as uninsured and under-insured adults [3,52,53]. The Inflation Reduction Act, passed in August 2022, also contained important also contained key provisions that (1) removed cost-sharing for all ACIP recommended vaccinations in Medicare Part D and (2) mandated Medicaid coverage of all ACIP recommended vaccinations with no cost-sharing for all adults enrolled in Medicaid [54]. A greater emphasis on public education to increase the awareness of these ‘safety net’ programs, which are designed to provide access to vaccination for individuals with a lower income, may improve vaccine uptake. Despite the existence of these safety-net programs, gaps still exist where some patients may experience direct out-of-pocket costs for vaccination services. Additionally, indirect costs associated with vaccination services such as transportation remain a patient barrier.

Childhood vaccination rates remain high in the US, but there are pockets of under-vaccination where there may be an increased risk of vaccine-preventable disease outbreaks among children and adults [55,56,57,58]. Notably, this resulted in the re-emergence of measles after it was considered eliminated in the US [58,59]. These pockets of under-vaccination may be fueled in part by increased vaccine hesitancy and access barriers, including issues with physical access to a vaccinator and insurance coverage [58,60,61,62].

Doctors’ offices and local pharmacies were the most trusted places for respondents or their children to receive vaccinations. Previous research supports these findings that individuals in the US prefer designated medical locations for receiving vaccinations, although logistical barriers such as access to available locations may influence vaccine uptake [63,64]. Furthermore, the finding that respondents were more likely to use a pharmacy to receive vaccines than to have their child vaccinated, reinforces the importance of pharmacies as sites for adult vaccination. A systematic literature review previously reported that pharmacies are capable of increasing adult vaccination rates due to their convenience and accessibility, but that political and organizational barriers need to be alleviated to drive vaccine uptake at these locations [65].

A limitation of this study was that it focused on a relatively small sample size of 5000 respondents from 10 states in the US, however, there are likely cultural differences across all 50 US states and Washington DC that may influence the outcomes. Therefore, the findings presented in this study may not be nationally representative and future studies should seek to explore perceptions across all 50 US states and Washington DC. Furthermore, due to the nature of the cross-sectional and online self-reported survey study design, the study may be affected by the inherent limitations of a survey, including self-reporting bias, recall bias, and the fact that the survey was only open to individuals without impediments such as a low level of literacy. This survey was conducted during the COVID-19 pandemic when attitudes surrounding vaccination were heightened and therefore the results may have been different had the study been conducted outside of the pandemic. The findings only reflect feelings towards the SARS-CoV-2 variants that were circulating at one point in time. Therefore, any variants that emerged after study completion are not captured in these results.

Future research should examine perceptions of vaccination across all 50 states to build a greater understanding of the perceptions of vaccination across the US. This future research may inform vaccine educational initiatives and vaccine policy to increase vaccine uptake and address issues that contribute to a lack of vaccine confidence. There is also an opportunity to further investigate the impact of access to information and immunization records on vaccine uptake, as this is a under-researched topic.

## 5. Conclusions

Our study highlights individual perceptions and beliefs around vaccines and vaccination in the US population. This study provides critical insights, outlining the perceived importance of state immunization programs, access to health and immunization records, and trust in healthcare providers, and highlights concerns about the safety of vaccines. These insights can be leveraged by public health professionals and policy stakeholders to aid in the development and implementation of strategies to build and strengthen vaccine confidence and increase equitable access to vaccination. Future research should examine perceptions of vaccination across all 50 states to build a greater understanding of these perceptions across the US. 

## Figures and Tables

**Table 1:** Perceived importance of state vaccination programs per age group for all respondents without religious objections to vaccination (n = 4917).

	Babies Aged 0–2 Years	Children Aged 3–8 Years	Children Aged 9–12 Years	Adolescents Aged 13–18 Years	Adults Aged 19–49 Years	Adults Aged 50–64 Years	Adults Aged 65 Years or Older
Very important	67%	72%	74%	73%	67%	71%	75%
Somewhat important	22%	21%	21%	21%	24%	21%	18%
Not very important	8%	5%	4%	4%	7%	6%	6%
Not at all important	4%	2%	2%	2%	2%	2%	2%

## Data Availability

Written requests can be sent to the corresponding author.

## Supplementary Materials

The following supporting information can be downloaded at: https://www.mdpi.com/article/10.3390/vaccines10091470/s1. File S1: Extended methods and results on the impact of COVID-19.
