# Supplementary material for: Attitudes and Beliefs around the Value of Vaccination in the United States
Source: Vaccines (Basel). 2022 Sep 5;10(9):1470. doi: 10.3390/vaccines10091470 (PMC9501875; doi:10.3390/vaccines10091470)
Supplement: Supplementary file 1 [file vaccines-10-01470-s001.zip › vaccines-1852019-supplementary.pdf]

Supplementary Materials: Extended methods and results on the impact of COVID-19

Methods

Respondents were presented with a series of questions for which they selected a response from a prespecified. Data collected included the impact of the COVID-19 pandemic on respondents' feelings towards vaccination and the impact of the COVID-19 pandemic on the likelihood of them seeking routine vaccinations for themselves or their children in the future.

Most survey questions encompassed vaccinations in general for all populations, with some targeting a particular age group. Most survey questions used a Likert scale whereby respondents were asked to select a response from the scale, for example, from "much more likely" to "much less likely". One question allowed for free text input, which asked respondents to describe the ways in which their views of vaccination had changed due to the COVID-19 pandemic.

Survey screener

BASE: TOTAL RESPONDENTS

RESP_AGE. What is your date of birth?

[YEAR DROPDOWN] [MONTH DROPDOWN]

[IF < 18 OR > 80, TERMINATE]

BASE: TOTAL RESPONDENTS

GENDER_NONBINARY_. Are you…?

**SR**	
1	Male
2	Female
3	Other
4	Prefer not to answer

BASE: TOTAL RESPONDENTS

QMktSize_US. Please insert your zipcode:

_____ **[ALLOW FOR 5 DIGITS]**

[HCAL_REGION1_Label_US [Hidden], TERMINATE IF NOT IN NJ, PA, OH, IL, TN, NC, LA, OK, NV OR ID]

BASE: TOTAL RESPONDENTS

KIDS02. How many children under the age of 18 are living in your household? Please reference only the children for which you are the parent or legal guardian. (If there are no children under 18 in your household, please type 0)

_______ **[RANGE 0-20]**

BASE: HAS CHILDREN (KIDS02 > 0)

KIDS3. How many children do you have in your household in each of the following age groups?

_______ Aged 0-11 **[RANGE 0-KIDS02]**

_______ Aged 12-18 **[RANGE 0-KIDS02]**

[TOTAL MUST SUM TO KIDS02]

BASE: TOTAL RESPONDENTS

USHHI3. Please indicate your **annual household income** before taxes.

[DROPDOWN]

_1 Less than $5,000

_2 $5,000-$9,999

_3 $10,000-$14,999

_4 $15,000-$19,999

_5 $20,000-$24,999

_6 $25,000-$29,999

_7 $30,000-$34,999

_8 $35,000-$39,999

_9 $40,000-$44,999

_10 $45,000-$49,999

_11 $50,000-$54,999

_12 $55,000-$59,999

_13 $60,000-$64,999

_14 $65,000-$69,999

_15 $70,000-$74,999

_16 $75,000-$79,999

_17 $80,000-$84,999

_18 $85,000-$89,999

_19 $90,000-$94,999

_20 $95,000-$99,999

_21 $100,000-$124,999

_22 $125,000-$149,999

_23 $150,000-$199,999

_24 $200,000-$249,999

_25 $250,000 or more

_99 Prefer not to answer

[TERM IF “PREFER NOT TO ANSWER”]

BASE: TOTAL RESPONDENTS

USO1ETH. Which of the following best describes you?

_1 White or Caucasian (not Hispanic or Latino)

_2 Black of African American (not Hispanic or Latino)

_3 Asian/Pacific Islander

_4 Native American, Alaska Native, Aleutian

_5 Hispanic or Latino (White or Caucasian)

_6 Hispanic or Latino (Black or African-American)

_7 Hispanic or Latino (all other races/multiple races)

_8 Other

_9 Prefer not to answer

[TERM IF “PREFER NOT TO ANSWER”]

BASE: TOTAL RESPONDENTS

S5. Which of the following best describes where you live? Please select one.

Major metropolitan area (one of the 20 largest cities, such as New York, Los Angeles, Chicago, Dallas)

A city that is not one of the top 20 largest US cities

Suburb (Outside a city)

Small town (a town of roughly 2,500 residents or more that is not considered a suburb)

Rural community (a town of less than roughly 2,500 residents)

BASE: TOTAL RESPONDENTS

S10. Which of the following best describes your health insurance coverage?

1	Insurance through my employer
2	Insurance through my spouse's employer
3	Insurance purchased through an exchange
4	Medicare
5	Medicaid
6	Other Insurance (e.g., VA, TriCare)
7	Unsure
8	I don't have health coverage of any kind

BASE: TOTAL RESPONDENTS

S20a. Have you ever received a vaccination for a vaccine preventable disease (i.e., flu, HPV, measles hepatitis, tetanus)?

Yes

No

Prefer not to answer **[TERM]**

BASE: HAVE NOT RECEIVED A VACCINATION (S20a = 2)

S25a. Which of the following are reasons you have objected to receiving vaccinations? Select all that apply.

Religious beliefs

Philosophical / personal beliefs

Other

Prefer not to answer **[TERM]**

BASE: HAVE NOT RECEIVED A VACCINATION (S20a = 2) AND NOT DUE TO RELIGIOUS REASONS (S25a ≠ 1)

S30a. Which of the following best describes your general attitude toward **adult vaccinations** (i.e., flu, HPV, measles hepatitis, tetanus)?

(*Select one*)

My attitude is favorable; I believe that adult vaccinations can play an important role in healthcare

I have no strong positive or negative attitudes toward adult vaccinations, or I am still deciding

My attitude is unfavorable; I do not support the idea of adult vaccinations **[TERMINATE]**

BASE: HAS NOT VACCINATED BUT DOESN'T HAVE UNFAVORABLE OPINION OF VACCINES (S30A = 1 OR 2)

S32a. Which of the following are factors that have influenced your decision to not receive vaccinations? Select all that apply.

[RANDOMIZES]

I have concerns about vaccine safety

I have concerns about how vaccines work

I do not trust governmental agencies

I need more information about vaccines

I am not clear on where to get vaccines

I have difficulty making time to get vaccinated

I do not have easy access to vaccines (due to location or transportation)

I am concerned about the potential cost

I don't know if my insurance covers vaccines

I don't have health insurance

I do not feel that they are necessary

I do not feel that I am at risk by not being vaccinated

Other (specify)

BASE: HAS CHILDREN (KIDS02 > 0)

S20b. Has your child/children ever received a vaccination for a vaccine-preventable disease (i.e., pertussis, hepatitis, HPV, measles)?

Yes

No

Prefer not to answer **[TERM]**

BASE: CHILD HAS NOT RECEIVED A VACCINATION (S20b = 2)

S25b. Which of the following are reasons you have objected to receiving vaccinations for your child/children? Select all that apply.

Religious beliefs

Philosophical / personal beliefs

Other

Prefer not to answer **[TERM]**

BASE: CHILD HAS NOT RECEIVED A VACCINATION (S20B = 2) AND NOT FOR RELIGIOUS REASONS (S25B ≠ 1)

S30b. Which of the following best describes your general attitude toward **childhood vaccinations** (i.e., flu, HPV, measles, hepatitis, tetanus)?

(*Select one*)

My attitude is favorable; I believe that childhood vaccinations can play an important role in healthcare

I have no strong positive or negative attitudes toward childhood vaccinations, or I am still deciding

My attitude is unfavorable; I do not support the idea of childhood vaccinations **[TERMINATE]**

BASE: CHILD IS NOT VACCINATED BUT DOESN'T HAVE UNFAVORABLE OPINION OF VACCINES (S30B = 1 OR 2)

S32b. Which of the following are factors that have influenced your decision to not have your child/children receive vaccinations? Select all that apply.

[RANDOMIZES]

I have concerns about vaccine safety

I have concerns about how vaccines work

I do not trust governmental agencies

I need more information about vaccines

I am not clear on where to get vaccines

I have difficulty making time to get vaccinated

I do not have easy access to vaccines (due to location or transportation)

I am concerned about the potential cost

I don’t know if my insurance covers vaccines

I don’t have health insurance

I do not feel that they are necessary

I do not feel that my child/children are at risk by not being vaccinated

Other (specify)

BASE: HAVE RECEIVED A VACCINATION (S20 = 1)

S35. Have you ever been vaccinated for any of the following?

(Select one for each)

ACROSS THE TOP:

Yes, before the age of 18

Yes, after the age of 18

No

Not sure

PN: RANDOMIZE

DOWN THE SIDE:

Flu (influenza)

Hepatitis A

Hepatitis B

Pneumonia/pneumococcal (disease)

Human Papillomavirus (HPV)

Meningitis/Meningococcal

Tetanus

Shingles

Full survey

Intro.

We are conducting research to understand the people’s views of vaccines and vaccination in the United States. The survey will take less than 20 minutes of your time.

The survey is completely confidential. None of your responses will be attributed to you, so please feel free to be open and honest with your views. During your participation, you do not have to answer a question if you do not wish to do so and you have the right to withdraw from the survey at any time.

The aggregated results of this survey will be used for market research and public relations purposes and will be published but your name will not be attributed in any way. All responses will be reported in aggregated or anonymous form. All personally identifiable information will be kept confidential. All data protection and privacy policy practices adhere to the most recent data protection regulations. We also work in accordance with healthcare guidelines set out by EphMRA and the BHBIA. Please see this Privacy Notice for further details.

Do you consent to participate in this research as set out above and in the privacy notice?

(Please select one answer only)

- o Yes, I wish to continue
- o No, I do not wish to continue **[TERMINATE]**

Adverse Event text

As the sponsor of the research is a pharmaceutical company, we are required to pass on to the pharmaceutical company, any details of adverse events/product complaints pertaining to their products that are mentioned during the interview. If this happens, we will need to collect details and report the event, even if you have already done so. You will be asked whether you consent to us passing your details to the pharmaceutical company's Quality Assurance (QA) department for their follow up, but you may choose to remain anonymous. This will have no impact on the confidentiality and anonymity associated with the interview itself and the chance of this happening is very low.

Are you happy to participate in this research as set out above?

(Please select one answer only)

- o Yes, I wish to continue
- o No, I do not wish to continue **[TERMINATE]**

MQB

VALUE OF VACCINES / VACCINE PERCEPTIONS

Thank you for agreeing to participate in this survey today. For the remainder of this survey, when you see the term 'vaccination' we are referring to the process whereby a person is given protection from an infectious disease, by the administration of a vaccine (i.e., flu, HPV, measles hepatitis, tetanus). We are interested in your opinions about vaccines **in general, not just the COVID-19 vaccine** unless otherwise stated in the question text.

BASE: TOTAL RESPONDENTS

Q5. How important do you think it is for your state to have a vaccination program that works well to prevent diseases?

Your state's health department or agency runs a state-wide vaccination program that uses programs and efforts based on scientific research to raise the number of residents who get vaccines.

**1** Not at all important	**2** Not very important	**3** Somewhat important	**4** Very important

BASE: NOT RELIGIOUS OBJECTOR (DOES NOT QUALIFY FOR QUOTA 8)

Q10. How important do you think it is for your state to have a vaccination program that works well to prevent diseases among each of the following age groups?

- Babies aged 0-2 years
- Children aged 3-8 years
- Children aged 9-12 years
- Adolescents aged 13-18 years
- Adults aged 19-49 years
- Adults aged 50-64 years
- Adults aged 65 years or older

**1** Not at all important	**2** Not very important	**3** Somewhat important	**4** Very important

BASE: NOT RELIGIOUS OBJECTOR (DOES NOT QUALIFY FOR QUOTA 8)

Q12. **[IF QUOTA4=1 "**How important do you think it is to have your child/children vaccinated against vaccine preventable diseases?**"]** **[IF QUOTA4=2 "**How important do you think it is to have children vaccinated against vaccine preventable diseases?**"]**

**1** Not at all important	**2** Not very important	**3** Somewhat important	**4** Very important

BASE: TOTAL RESPONDENTS

Q15. Using the scale below, please indicate how much do you agree with each statement about vaccines.

I) **Health [PN: SHOW HEADING; RANDOMISE STATEMENTS WITHIN]**

- Vaccines help prevent disease for me

- Vaccines help prevent disease for children
- Vaccines help improve the quality of life of my community, such as being able to spend more time with family and friends
- Getting vaccinated helps to protect everyone from infectious disease
- The benefits of vaccines outweigh the risks

II) **Society [PN: SHOW HEADING; RANDOMISE STATEMENTS WITHIN]**

- Vaccines reduce the impact of infectious diseases for all of society
- Vaccines have the potential to improve life-expectancy
- Vaccinations are an important way to protect people around me
- Getting vaccinated against vaccine preventable diseases is a collective responsibility for all members of a society

III) Economy [PN: SHOW HEADING; RANDOMISE STATEMENTS WITHIN]

- In the long run, vaccines help to save on healthcare costs for individuals
- In the long run, vaccines help to save costs for the government and the healthcare industry
- Vaccinating the population can lead to productivity gains for individuals
- Vaccinating the population can lead to productivity gains for society in general
- Vaccinations help to minimize the economic impact of disease on families
- It is cheaper to prevent disease than treat it

[TOP, COLUMNS]

Strongly agree **[SHOW ON RIGHT]**

Agree

Disagree

Strongly disagree **[SHOW ON LEFT]**

BASE: TOTAL RESPONDENTS

Q25. How confident are you in the science behind vaccines, such as how they are made and how they work?

**1**<br>Not at all confident	**2**<br>Not very confident	**3**<br>Somewhat confident	**4**<br>Very confident

COVID IMPACTS

BASE: RECEIVED A VACCINE (S20A = YES)

Q100**.** How many doses of the COVID-19 vaccine have you received?

1 dose of a vaccine that requires 2 doses

1 dose of a vaccine that only requires 1 dose

2 doses of a vaccine that requires two doses

None, but I plan to get the vaccine

None, and I do not plan to get the vaccine

BASE: NOT PLANNING ON GETTING COVID VACCINE (Q100 = 4)
Q110. For which reasons are you not planning on receiving the COVID-19 vaccine? Select all that apply.

[RANDOMIZE, ANCHOR OTHER]
I don't think it is effective (works well to prevent COVID-19 infection)
I don't think it is safe
I don't think there is enough long-term data/information to make an informed decision
I have already had COVID-19 and do not think I need to be vaccinated as well
I don't think the risk from COVID-19 is worth getting a vaccine
I am healthy and do not need the vaccine
It is not needed
Other (specify)

BASE: TOTAL RESPONDENTS
Q115. How has the COVID-19 pandemic impacted your feelings towards vaccinations **in general**?

Much more positive
Somewhat more positive
About the same – no impact
Somewhat more negative
Much more negative
Not sure

BASE: IF VIEWS HAVE CHANGED (Q115 ≠ ABOUT THE SAME)
Q120. How did your views of vaccinations change due to the COVID-19 pandemic?

[OPEN TEXT BOX, MIN 10 CHARACTERS]

BASE: TOTAL RESPONDENTS

Q125. How has the COVID-19 pandemic impacted your likelihood to receive future routine vaccinations for other vaccine preventable diseases?

Much more likely
Somewhat more likely
About the same – no impact on how likely I am to get future routine vaccinations
Somewhat less likely
Much less likely
Not sure

BASE: HAS CHILDREN AGED 12-18 (KIDS3_2 > 0) AND HAVE RECEIVED A VACCINE BEFORE (S25B = YES)

Q130. You mentioned you have children aged 12-18. How many doses of the COVID-19 vaccine have your children received?

1 dose of a vaccine that requires 2 doses

2 doses (all doses needed)

None, but I plan to get them the vaccine

None, and I do not plan to get them the vaccine

BASE: RESPONDENTS WHO'S CHILDREN ARE NOT VACCINATED (Q130 = 4)
Q135. Why have you decided not to have your children get the COVID-19 vaccine?

[RANDOMIZE, ANCHOR OTHER]
I did not know my children (over the age of 12) could receive the vaccine
I don't think it is effective (works well to prevent COVID-19 infection)
I don't think it is safe
I don't think there is enough long-term data/information to make an informed decision
My child/children have already had COVID-19 and do not think they need to be vaccinated
I don't think the risk from COVID-19 is worth getting a vaccine
My children are healthy and do not need the vaccine
It is not needed
Other (specify)

BASE: TOTAL RESPONDENTS

Q140. How has the COVID-19 pandemic impacted your likelihood to have **your children** receive future vaccinations for vaccine preventable diseases?

Much more likely
Somewhat more likely
About the same – no impact on how likely I am get them future vaccines
Somewhat less likely
Much less likely
Not sure

BASE: IF VIEWS HAVE CHANGED (Q140 ≠ ABOUT THE SAME)
Q145. How did your views of vaccinations for **children** change due to the COVID-19 pandemic?

[OPEN TEXT BOX, MIN 10 CHARACTERS]

VALUE OF HEALTH RECORDS

BASE: TOTAL RESPONDENTS
Q200. How important is it for you to have access to your health records **in general** for future health planning?

1<br>Not at all important	2<br>Not very important	3<br>Somewhat important	4<br>Very important

BASE: NOT RELIGIOUS OBJECTOR (DOES NOT QUALIFY FOR QUOTA 8)
Q205. How important is it for you to have access to your immunization (vaccination) records **in general** for future health planning?

1<br>Not at all important	2<br>Not very important	3<br>Somewhat important	4<br>Very important

BASE: NOT RELIGIOUS OBJECTOR (DOES NOT QUALIFY FOR QUOTA 8)
Q210. How important is it for states **in general** to have immunization information systems?

Immunization information systems (IISs), also called Immunization Registries, are confidential, computerized databases that track all vaccine doses given by healthcare providers to residents in a state.

1<br>Not at all important	2<br>Not very important	3<br>Somewhat important	4<br>Very important

BASE: NOT RELIGIOUS OBJECTOR (DOES NOT QUALIFY FOR QUOTA 8)
Q215. Using the scale below, please indicate how much do you agree with the following statement.

Immunization (vaccination) records and immunization information systems (IISs) play an important role in managing and stopping the spread of diseases that can be prevented by getting vaccines.

Strongly agree **[SHOW ON RIGHT]**

Agree

Disagree

Strongly disagree **[SHOW ON LEFT]**

ACCESS PRIORITIZATION

NOT RELIGIOUS OBJECTOR (DOES NOT QUALIFY FOR QUOTA 8)
Q300. Where do you **normally** receive vaccinations? Select all that apply.

[RANDOMIZE, ANCHOR OTHER]
Through my doctor’s office
Through a local pharmacy (e.g CVS, Walmart, Walgreens)
Through a community clinic

Through a school clinic
Through an employer-based clinic
At a hospital
Other (specify)

BASE: HAS CHILDREN AGED 12-18 (KIDS3_2 > 0) AND NOT RELIGIOUS OBJECTOR (DOES NOT QUALIFY FOR QUOTA 8)
Q302. Where does your **child/children normally** receive immunizations? Select all that apply.

[RANDOMIZE, ANCHOR OTHER]
Through my doctor's office
Through a local pharmacy (e.g CVS, Walmart, Walgreens)
Through a community clinic
Through a school clinic
Through an employer-based clinic
At a hospital
Other (specify)

NOT RELIGIOUS OBJECTOR (DOES NOT QUALIFY FOR QUOTA 8)
Q305. Where would you **most prefer** to get vaccines? Select one.

[SHOW IN SAME ORDER SHOWN IN Q300]
Through my doctor's office
Through a local pharmacy (e.g CVS, Walmart, Walgreens)
Through a community clinic
Through a school clinic
Through an employer-based clinic
At a hospital
Other (specify)

BASE: NOT RELIGIOUS OBJECTOR (DOES NOT QUALIFY FOR QUOTA 8)
Q310. Where do you **typically** get information about vaccines? Select all that apply

[RANDOMIZE, ANCHOR OTHER]
My doctor / Healthcare professional
Friends
Family
Employer
Television News
Newspapers
Online News Sources
Social Media
Political Leaders
Religious Leaders
Business and Community Leaders
Online information sources and government agencies such as the Centers for Disease Control (CDC)
Other (specify)

BASE: TOTAL RESPONDENTS

Q315. Using the scale below, please indicate how much do **you personally trust** information about immunizations/vaccines from each of the following sources?

[SHOW IN SAME ORDER SHOWN IN Q310]

My doctor / Healthcare professional
Friends
Family
Employer
Television News
Newspapers
Online News Sources
Social Media
Political Leaders
Religious Leaders
Business and Community Leaders
Online information sources and government agencies such as the Centers for Disease Control (CDC)
Other (specify)

SCALE [TOP]

Strongly trust **[SHOW ON RIGHT]**

Slightly trust

Slightly mistrust

Strongly mistrust **[SHOW ON LEFT]**

BASE: TOTAL RESPONDENTS

Q320. For each of the following sources, please tell me if you think the **general public generally trusts** them to tell the truth, or not. Select one response for each source.

My doctor / Healthcare professional
Friends
Family
Employer
Television News
Newspapers
Online News Sources
Social Media
Political Leaders
Religious Leaders
Business and Community Leaders
Online information sources and government agencies such as the Centers for Disease Control (CDC)
Other (specify)

SCALE [TOP]

Strongly trust **[SHOW ON RIGHT]**

Slightly trust

Slightly mistrust

Strongly mistrust **[SHOW ON LEFT]**

BASE: NOT RELIGIOUS OBJECTOR (DOES NOT QUALIFY FOR QUOTA 8)
Q325. Using the scale below, how **important** are each of the following when receiving a vaccination?

[RANDOMIZE ROWS]
Location (where you get the vaccine)
Hours available
Provider type (i.e primary care doctor, specialist doctor, pharmacist, etc.)
Insurance coverage
Costs you may have to pay
Ability to get the vaccine during a yearly checkup or other visit for another health problem

**1** Not at all important	**2** Not very important	**3** Somewhat important	**4** Very important

DEMOGRAPHICS

Lastly, we have a few final questions to learn a bit more about you.

BASE: TOTAL RESPONDENTS
USEDU3. What is the highest degree or level of school you have completed? Select only one

_1 Grade 4 or less
_2 Grade 5 to 8
_3 Grade 9 to 11
_4 Grade 12 (no diploma)
_5 Regular High School Diploma
_6 GED or alternative credential
_7 Some college credit, but less than 1 year
_8 1 or more years of college credit, no degree
_9 Associate's degree (AA, AS, etc)
_10 Bachelor's degree (BA, BS, etc.)
_11 Master's degree (MA, MS, MBA, etc.)
_12 Professional degree (MD, DDS, JD, etc.)
_13 Doctorate degree (PhD, EdD, etc.)

BASE: TOTAL RESPONDENTS
D5. What best describes your politics?

Strong Democrat

Moderate Democrat
Lean Democrat
Lean Republican
Moderate Republican
Strong Republican
Independent
Other
Not sure
Prefer not to answer

BASE: TOTAL RESPONDENTS

D10. At the present time, are you…? Please select one.

Working part-time

Working full-time

Temporarily unemployed

Disabled or unable to work

Choose not to work

Retired from a paying job

Prefer not to answer

BASE: TOTAL RESPONDENTS
D15. How confident are you filling out medical forms by yourself?

Extremely
Quite a bit
Somewhat
A little
Not at all

END OF QUESTIONNAIRE – CLOSE AND THANK

Results

Impact of COVID-19 pandemic on perceptions of vaccination in general

An overview of respondent characteristics is shown in Table S1. Nationwide, 40% of respondents reported that the COVID-19 pandemic made them feel more positive towards vaccinations in general, while 45% reported no impact, and 15% reported that the pandemic made them feel more negative towards vaccinations. Respondents in the Northeast were the most likely to report that the pandemic had a positive impact on their perceptions of vaccines (45%), while the respondents in the West and South-Central were most likely to report that the pandemic had a negative impact on their perceptions of vaccines with 19% and 18%, respectively. By state, respondents in New Jersey were most likely to report a positive impact (49%), while respondents in Idaho were most likely to report a negative impact (22%) on their perception of vaccination.

All respondents were asked to describe the ways in which their perceptions had changed (n=2,773). Among all participants, the most common theme identified was that 21% indicated a change to viewing vaccines as “necessary” or “important” and something that all individuals should receive (Figure S2). Concerns about the safety of vaccines, that they are potentially “not researched or tested well” or were “rushed”, were identified by 8%. Individual state analysis revealed that safety concerns were mentioned most often by respondents in Idaho (16%).

In all regions, 53% of respondents reported that the COVID-19 pandemic did not influence the likelihood of them to receive future routine vaccinations for other vaccine-preventable diseases; with 37% indicating they were more likely to receive future routine vaccinations, 8% were less likely to receive future routine vaccinations, and 2% were unsure; Figure S3. The greatest proportion of respondents who were now more likely to receive future routine vaccinations was 40% in the Northeast, while the greatest proportion of respondents who were less likely to receive future routine vaccinations was 11% in the West. On a state level, the greatest proportion of respondents who were more likely to receive future routine vaccinations was 43% in Louisiana, while the greatest proportion of respondents who were less likely to receive future routine vaccinations was 13% in Idaho.

Impact of COVID-19 pandemic on perceptions of vaccination for children

Similar to the findings regarding vaccinations in general, most respondents reported that the COVID-19 pandemic had no impact on the likelihood of them having their children receive future routine vaccinations for other vaccine-preventable diseases (49%). Nationally, 39% reported that they were now more likely to have their children receive future routine vaccinations, 10% reported that they were less likely to have their children receive future routine vaccinations, and 2% were unsure. The greatest proportion of respondents who were more likely to have their children receive future routine vaccinations was in the Northeast with 48%. The greatest proportion of respondents who were less likely to have their children receive future routine vaccinations was in the West with 13%.

All respondents were asked to describe the ways in which their perceptions had changed (n=4,390; Figure S4). Nationwide, respondents most frequently reported that “nothing fundamentally changed” regarding their views on vaccination for their children (39%). The second most common mention was that respondents viewed vaccinations as “necessary” or “important” and expressed that all individuals should be vaccinated.

Tables and figures

Tables

Table S1. Demographics for all respondents (n=5,000)

	Approximate proportion of respondents (%)
**Age**	
Mean (years)	53
Male	43%
Female	56%
Other	1%
Prefer not to say	<1%
**Ethnicity**	
Caucasian	80%
Black or African American	8%
Latinx	6%
Other	5%
**Location**	
Major metropolitan area[a]	10%
A city[b]	19%
Suburb	40%
Small town[c]	15%
Rural community[d]	16%
**Annual household income ($USD)**	
<30,000	25%
30,000–50,000	21%
50,000–75,000	20%
75,000–100,000	13%
100,000–150,000	11%
≥150,000	7%
Prefer not to answer	4%
**Insurance coverage**	
Public	40%
Private	47%
Unsure/other	7%
None	5%
**Vaccination status**	
Had received a vaccination for a vaccine-preventable disease	95%
Had not received a vaccination for a vaccine-preventable disease	5%
**Children's vaccination status**	
Had received a vaccination for a vaccine-preventable disease	91%
Had not received a vaccination for a vaccine-preventable disease	9%

[a] One of the 20 largest cities, such as New York, Los Angeles, Chicago, or Dallas.
[b] Not one of the top 20 largest US cities.
[c] A town of roughly 2,500 residents or more that is not considered a suburb.
[d] A town of less than roughly 2,500 residents.

Figures

Figure S1. Map of the 10 states included in the study[a]

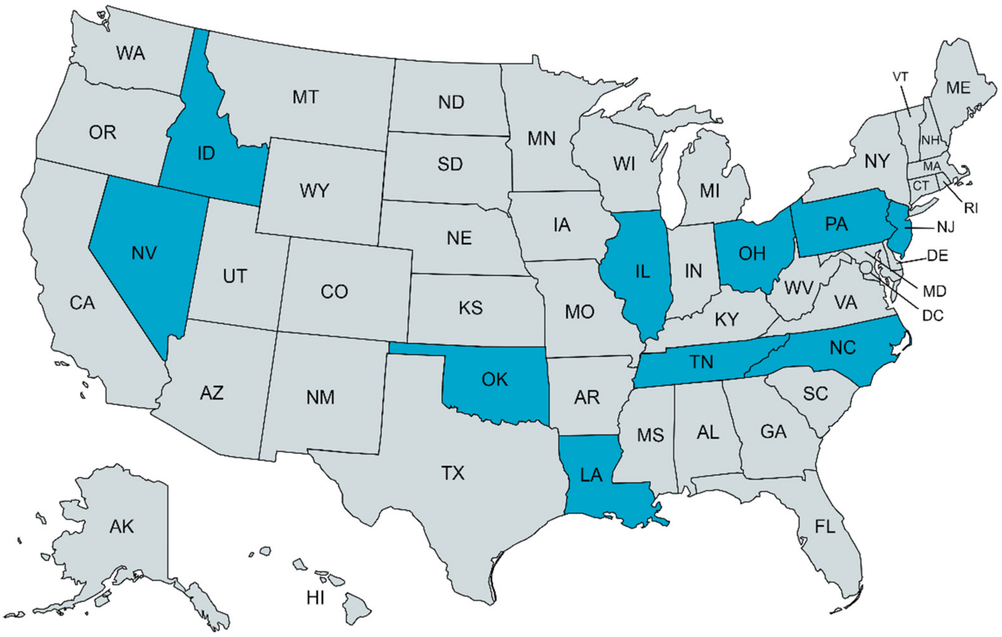

[a]Blue shading indicates states that were included in this study.

Figure S2. Top five themes among respondents that reported if their views of vaccination had changed as a result of the COVID-19 pandemic (n=2,773)[a]

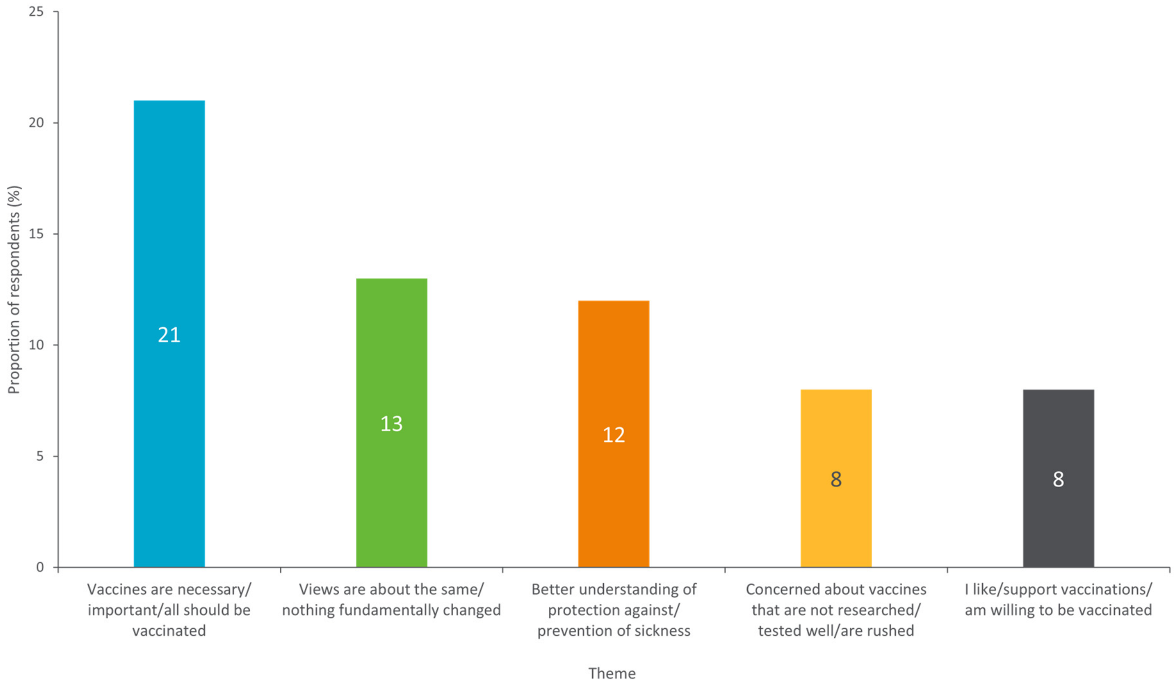

[a]Results are for respondents across the 10 US states captured.

Figure S3. Percent of respondents that reported the impact of the COVID-19 pandemic on the likelihood of receiving future routine vaccinations (n=5,000)[a]

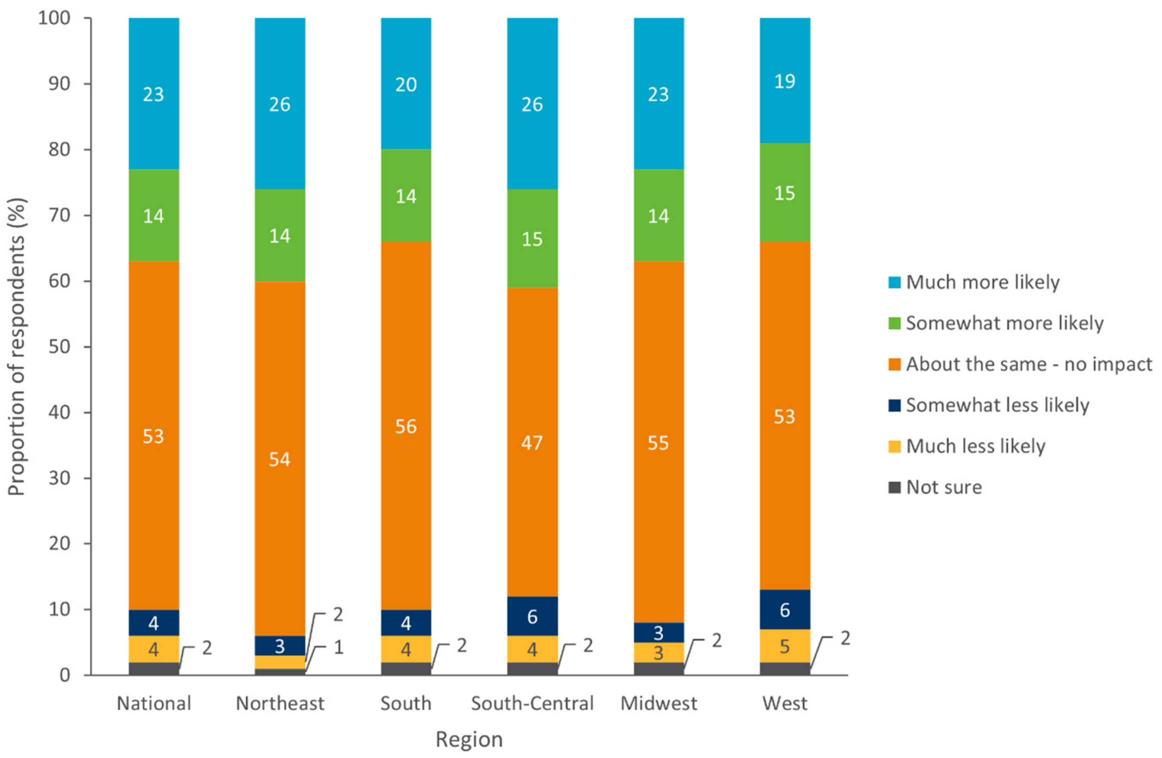

[a] Results split by US region.

Figure S4. Common themes among respondents who reported if they were more or less likely to have their children vaccinated against other vaccine-preventable diseases as a result of the COVID-19 pandemic (n=4,390)[a,b]

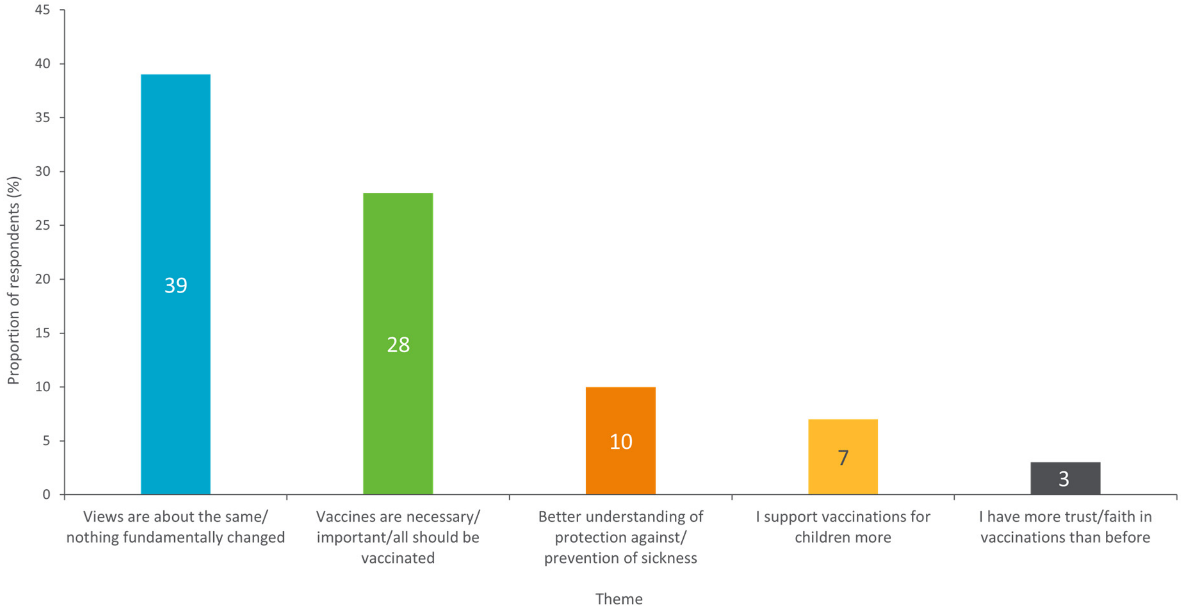

[a]Results are for respondents across the 10 US states captured.

[b]This sample included both respondents with children and respondents without children.
